# Dizygotic Dichorionic Triamniotic Triplet Pregnancy Delivered at Full Term: An Out of Box Presentation of Triplet—A Case Report from Ethiopia

**DOI:** 10.1155/2018/5760147

**Published:** 2018-08-08

**Authors:** Melese Gezahegn Tesemma, Mikiyas Tadesse Yadeta

**Affiliations:** Department of Obstetrics and Gynecology, Jimma University Medical Center (JUMC), Jimma, Ethiopia

## Abstract

The incidence of triplet is raising several hundred percent due to wide availability of fertility therapies. It is associated with different perinatal and maternal complications. The average duration of gestation and birth weight for triplet are 32.5 weeks and 1735 grams (total weight of triplet set being about 5.2kg), respectively. However, it is not uncommon to find the rarest situations in obstetrics. Here we present a case of triplet set born from gravida 5 para 4 mother at full term (GA of 39 weeks and 3 days), dated from reliable last normal menstrual period (LNMP). Each triplet has weight comparable to average weight of singleton at term. Triplets A, B, and C weigh 2.8 kg, 3 kg, and 3.2 kg, respectively. The triplet set weigh 9 kg in aggregate. Surprisingly the neonates have no perinatal complication, but the mother developed postpartum hemorrhage secondary to uterine atony. All individual triplets are large for gestational age on adjusted fetal weight standard of triplet from singleton's growth curve. Such type of triplet outcome is unique in its presentation and has never been reported in scientific literature as to the knowledge of the authors. Thus, we can consider it as “an out of box presentation of triplet”. This initiates us to report the case.

## 1. Introduction

The incidence of triplet and higher order multifetal gestation is rising since 1980, primarily due to the increasingly widespread availability of infertility therapies and postponing conception to older age [1]. The incidence of triplet delivery in humans is approximately one in 6400 pregnancies and the monochorionic triplet subset may occur only once in 100,000 births [2]. Triplet pregnancy is associated with significantly increased risks of maternal and neonatal morbidity which includes preterm labor, prematurity, anemia, gestational diabetes, preeclampsia, amniotic fluid abnormalities antepartum hemorrhage, postpartum hemorrhage, still births, and perinatal deaths [3].

The average duration of gestation at delivery for singletons, twins, and triplets is 39, 35, and 32 weeks, respectively [1]. In one large series of 198 triplet pregnancies, proportion of delivery that occurred at GA of > 37 weeks, at 32 to 37 weeks, at 29 to 31 weeks, and < 29 weeks were 5 %, 75 %, 13%, and 7 %, respectively [4]. The mean GA and mean birth weight at delivery for triplets are 32.5 weeks and 1735 grams, respectively, as compared to singletons with 39.0 weeks and 3357 grams [5].

In one study, it is found that about 95 % and 35 % of triplets have low birth weight (LBW), i.e., < 2500g, and very low birth weight (VLBW), i.e., < 1500g, respectively. By comparison, the rates of LBW and VLBW in singletons are only 6.5% and 1.1 %, respectively. The average twin weighs 960 grams less than the average singleton at birth while triplets typically weigh about one-half that of infants in singleton deliveries [1].

## 2. Case Report

A 35-year-old gravida five para four mother with gestational age of 39 weeks and 3 days, dated from reliable LNMP, is admitted to Madawalabu general hospital. She is referred from health center with a diagnosis of twin gestation for better management. Her antenatal follow-up was at health center five times. She finished her immunization against tetanus and was taking her iron supplementation regularly. Her blood group & Rh is AB+ and preoperative hematocrit is 30% while other tests are normal. On presentation to the hospital, she has no danger signs of pregnancy like vaginal bleeding, headache, blurring of vision, and passage of liquor. She has no pushing down pain as well. She has neither personal/family history of multiple gestations nor history of taking fertility drugs. She has no personal or family history of diabetes, obesity, hypertension, and other chronic medical illnesses.

She noticed undue enlargement of abdomen and excessive increment in fetal kicks in the last trimester. She has got difficulty in undergoing daily routines during the last one month and difficulty in walking comfortably for last two weeks. Moreover she leaves her bed only with family support for the last one week due to abdominal heaviness and significantly increased body weight. Her prepregnancy weight and height were 74kg and 170cm making prepregnancy BMI of 25.6 kg/m^2^ but the current weight is 98kg. The pregnancy is planned, wanted, and supported.

Upon examination, general appearance is well looking. Vital signs are BP=100/70mmHg, PR=98bpm, RR=22bpm, and T^0^=36.6°C. On abdominal examination, abdomen is grossly distended, SFH measures 46cm with tape meter, multiple fetal poles felt. Fetal heart beat heard at multiple sites. She has no uterine contraction. No abnormality detected in other systems. Up on scanning with obstetric U/S, there is triplet intrauterine pregnancy, two fundal placentas with two visible dividing membrane. Triplet A is breech in its presentation, with aggregate GA of 37 weeks + 4 days, EFW 2708g, and biophysical profile (BPP) of 8/8; triplet B is breech, with AGA 37 weeks + 1 day, EFW 2918 g, and BPP of 8/8; and triplet C has transverse lie, with AGA 38 weeks + 2 days, EFW 3104g, and BPP of 8/8.

**Figure 1:**
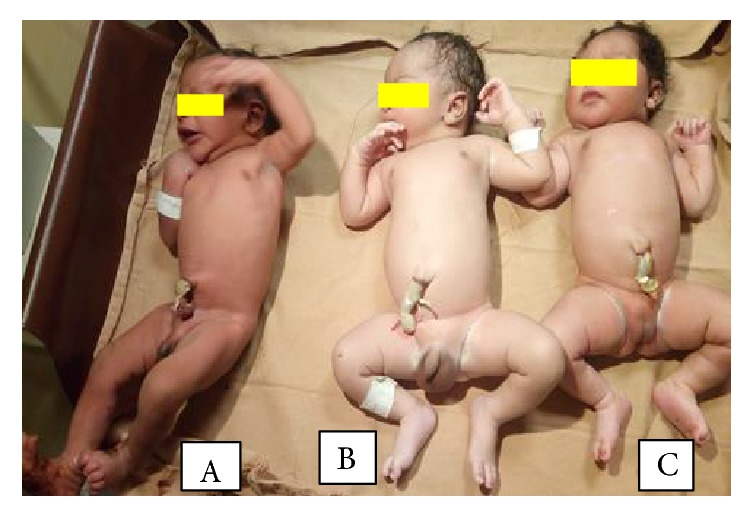
Picture showing triplet sets at the time of delivery. Triplets A to C (left to right).Triplet A: male weighing 2.8 Kg. Triplet B: male weighing 3 Kg. Triplet C: female weighing 3.2 Kg.

**Figure 2:**
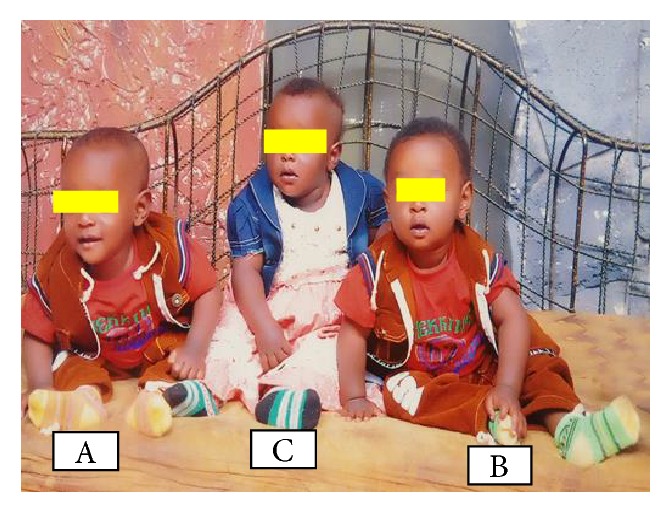
Picture showing the well-grown triplet infants at their 10^th^ months of life.

With the final diagnosis of full term + triplet pregnancy + mild anemia, she was prepared for elective C/S. Lower uterine segment transverse C/S done to effect the delivery of an alive triplet sets triplet A, male weighing 2800 g, triplet B, male weighing 3000 g, and triplet C, female weighing 3200g, all of them with APGAR score of 8/10 and 9/10 at first and fifth minutes of life. The first two triplets share the same placenta weighing 600g while the 3^rd^ female triplet has its own placenta weighing 480g but all of them having their own amniotic sac. (Figure 1). Following delivery of placentas and uterine closure, the uterus became atonic for which we put on oxytocin drip and sublingual 600 *μ*g of misoprostol. Fortunately, the uterus responded for oxytocin drip and sublingual misoprostol without requiring further management. Estimated blood loss is about 1300ml. Uterus was massaged every 15 minutes for first 2 hours. Her postop hematocrit was 24 % for which she was given therapeutic iron sulfate. Postoperative period was smooth and the mother with all the triplet was discharged on the 3^rd^ postop day. The infants were followed for 10 months at which time no problem with physical growth is observed; rather apparent normal neurological development is seen. All of them are in good health status as well. The picture below was taken on 10^th^ month of their life (Figure 2).

## 3. Discussion

This is a case of spontaneous triplet pregnancy, which is not due to ovulation induction at GA of 39 weeks and 3 days. The presence of two placenta at delivery, one separate placenta supplying female fetus and the other being shared by two male fetuses, but all of them having their own amnion, makes it dichorionic, dizygotic, triamniotic triplet set.

The growth of triplet and singleton is almost similar up to 26 weeks. From 26 to 32 weeks, the average triplet newborn has a weight corresponding to approximately the 30th percentile level compared with singletons. After 32 weeks, the growth curve of singleton gets steeper and that of triplet birth weights falls progressively behind those of singletons, reaching the 10th percentile at 38 weeks. Thus, the mean birth weight of triplets was slightly below the 10th percentile for singletons at GA of 38 weeks and beyond [6]. Accordingly, in our case not only the largest, but also the smallest triplet is above this adjusted fetal weight standard for triplet and thus, large for gestational age.

There are several factors that affect outcome in high order multiple gestations. These are maternal height, parity, number of live fetuses, placentation, and discordant growth. Height of the mother >165cm, multiparty, live sets of multiple gestation, and having one placenta for each fetus will improve the birth outcome by having greater birth weight and GA at birth [7]. Death of one or two fetuses in triplet set has resulted in very preterm delivery at 31 weeks and VLBW (1380 grams) in one published case report [8]. In our case, the mother is multiparous and 170cm tall while all the fetuses are alive, dichorionic, and triamniotic all of which are favoring good neonatal outcome.

Discordant growth among fetuses comprising the triplet gestation is common. Birth weight discordance is significantly associated with both fetal and neonatal mortality. Approximately 20% of triplet sets experience a birth weight discordance of 25 – 35% and nearly 10% experience severe forms of discordance (>35%) [9]. It is only 12.5% and 6.2% in our case for the other two triplets as compared to biggest triplet. Weight gain and the pattern of weight gain during the pregnancy are important predictors of fetal weight in triplets. Better intrauterine growth for gestational age is achieved in triplet gestations with maternal weight gains of >1.5 lbs./week before 24 weeks' gestation [10]. In our case, there was no significant birth weight discordance and the mother gained about 24kgs during this pregnancy predicting good neonatal outcome.

Deciding upon an upper gestational age for elective delivery is not usually an issue with triplet gestations; a large epidemiologic analysis found that only 16 percent remain undelivered at 36 weeks of gestation. The nadir of perinatal mortality for triplet pregnancies occurs at approximately 35 weeks [11]. In our case, GA at delivery was prolonged for triplet gestation. This is because the diagnosis of triplet was missed till the date of admission to the hospital as her antenatal care was at health center by midwifes where she was diagnosed to have twin gestation. Although there is no standard recommended time for elective delivery, many experts agree that it is reasonable to offer delivery of uncomplicated triplets anytime between 35 and 36 weeks as mentioned in up-to-date electronic reading material. Our case could have undergone elective caesarean delivery at GA of no later than 37 weeks had it been possible to diagnose triplet earlier in the gestation and her antenatal follow-up was in our hospital. Surprisingly, our case carried pregnancy to full term without any perinatal complication apart from maternal discomfort she faced during the latter two weeks.

## 4. Conclusion

In our case, being multipara, having nonmonochorionic placentation, absence of fetal growth discordance, maternal height of 170cm, and excessive weight gain of 24kgs during pregnancy might have contributed for this prolonged GA at birth, and large for gestational age triplet set. Delivery of triplet set at full term (39 weeks + 3 days), with individual triplet weight comparable to singleton weight at term, but with no adverse perinatal outcome, is unique in its type and has never been reported in scientific literature as to the knowledge of authors. Thus, we can consider it as “an out of box presentation of triplet”.
